# Comparison of PD-L1 protein expression between primary tumors and metastatic lesions in triple negative breast cancers

**DOI:** 10.1136/jitc-2020-001558

**Published:** 2020-11-25

**Authors:** Mariya Rozenblit, Richard Huang, Natalie Danziger, Priti Hegde, Brian Alexander, Shakti Ramkissoon, Kim Blenman, Jeffrey S Ross, David L Rimm, Lajos Pusztai

**Affiliations:** 1 Department of Medical Oncology, Yale University School of Medicine, New Haven, Connecticut, USA; 2 R&D, Foundation Medicine Inc, Cambridge, Massachusetts, USA; 3 Department of Pathology and Comprehensive Cancer Center, Wake Forest School of Medicine, Winston-Salem, North Carolina, USA; 4 Pathology and Urology, Upstate Medical University, Syracuse, New York, USA; 5 Department of Pathology, Yale University School of Medicine, New Haven, Connecticut, USA

**Keywords:** B7-H1 antigen, breast neoplasms, tumor microenvironment

## Abstract

Programmed Death Ligand 1 (PD-L1) positivity rates differ between different metastatic sites and the primary tumor. Understanding PD-L1 expression characteristics could guide biopsy procedures and motivate research to better understand site-specific differences in the tumor microenvironment. The purpose of this study was to compare PD-L1 positivity on immune cells and tumor cells in primary and metastatic triple negative breast cancer (TNBC) tumors. Retrospective study utilizing the PD-L1 database of Foundation Medicine containing the SP142 companion diagnostic immunohistochemistry assay (SP142 CDx) and Food and Drug Administration guidelines for scoring. 340 TNBC cases (179 primary tumors and 161 unmatched metastatic lesions) were evaluated. The primary outcome measures were PD-L1 positivity rates in immune cells and tumor cells. χ^2^ test was used for comparisons. Spearman’s correlation coefficient was used for correlations. More primary tumors were positive for PD-L1 expression on immune cells than metastatic lesions (114 (63.7%) vs 68 (42.2%), p<0.0001). This was driven by the lower PD-L1 positivity rates in skin (23.8%, 95% CI: 8.22% to 47.2%), liver (17.4%, 95% CI: 5.00% to 38.8%) and bone (16.7%, 95% CI: 2.10% to 48.4%) metastases. Lung (68.8%, 95% CI: 41.3% to 90.0%), soft tissues (65.2%, 95% CI: 42.7% to 83.6%) and lymph nodes (51.1%, 95% CI: 35.8% to 66.3%) had PD-L1 % positivity rates similar to primary tumors. PD-L1 expression was rare on tumor cells in both the breast and metastatic sites (8.3% vs 4.3%, p=0.13). The rate of PD-L1 positivity varies by metastatic location with substantially lower positivity rates in liver, skin and bone metastases compared with primary breast lesions or lung, soft tissue or lymph node metastases. This difference in PD-L1 positivity rates between primary tumors and different metastatic sites should inform physicians when choosing sites to biopsy and suggests a difference in the immune microenvironment across metastatic sites.

## Background

In March 2019, atezolizumab, a monoclonal antibody targeting Programmed Death Ligand 1 (PD-L1), was approved by the US Food and Drug Administration in combination with nab-paclitaxel as first-line therapy for unresectable or metastatic PD-L1 positive triple negative breast cancer (TNBC) based on results of the IMpassion-130 trial. PD-L1 positivity was defined as tumors that express PD-L1 on immune cells that cover 1% or more of the tumor area using the SP142 companion diagnostic immunohistochemistry assay (SP142 CDx). The trial assessed PD-L1 expression on primary tumors (60% of patients) and metastatic lesions (40% of cases), and efficacy of treatment appeared to be similar, although not formally compared, regardless of whether primary tumor or metastases were used to define PD-L1 positivity.^1 2^ Several recent studies that compared small cohorts of metastatic and primary lesions, as well as paired metastatic and primary breast tumors from the same patient, suggested substantial heterogeneity in tumor infiltrating lymphocyte count, immune gene expression and PD-L1 protein expression across different metastatic sites and between primary breast cancers and metastases.^3–6^ Understanding the frequency of PD-L1 positivity rates across different tissue sites can indicate differences in the immune microenvironment and may also guide biopsy site selection. Using the Foundation Medicine PD-L1 immunohistochemistry (IHC) database, we present PD-L1 positivity data by metastatic tissue site of origin. Immune fitness changes during aging,^7^ therefore we also assessed association between age and PD-L1 positivity.

## Methods

Approval for this study, including a waiver of informed consent and a Health Insurance Portability and Accountability Act (HIPAA) waiver of authorization, was obtained from the Western Institutional Review Board (Protocol No. 20152817). A retrospective data analysis of the Foundation Medicine clinical database was conducted on 340 cases of TNBC that were assessed for PD-L1 expression in the context of routine care. These cases were the first 340 cases that were stained at Foundation Medicine with the SP142 CDx assay using the CDx scoring system for TNBC in 2019 following the CDx approval for atezolizumab with nab-paclitaxel in TNBC. Only cases that were confirmed to be TNBC based on the review of the accompanying pathology report and/or FoundationOne CDx ERBB2 amplification results were included in this cohort.

SP142 CDx PD-L1 positivity was determined by IHC following the manufacturer’s recommendations. On any given day, scoring was performed by one of six pathologists who were each trained in PD-L1 CDx interpretation, and borderline cases were reviewed by at least two pathologists to arrive at a consensus. Results are reported as percent of PD-L1-stained immune cells in the tumor area. A tumor was considered PD-L1 positive if ≥1% immune cells stained positive with PD-L1. As an exploratory analysis, PD-L1 positivity of tumor cells was also assessed. PD-L1 percent positive staining results are reported as means with 95% CIs. The proportion of PD-L1 positive and negative immune cells and tumors cells in primary tumors versus metastatic sites was compared using χ^2^ test. Correlation between patient age at the time of testing and PD-L1 positivity was assessed using the Spearman’s correlation coefficient. All data analysis was conducted using Prism V.8.

## Results

**Figure 1:**
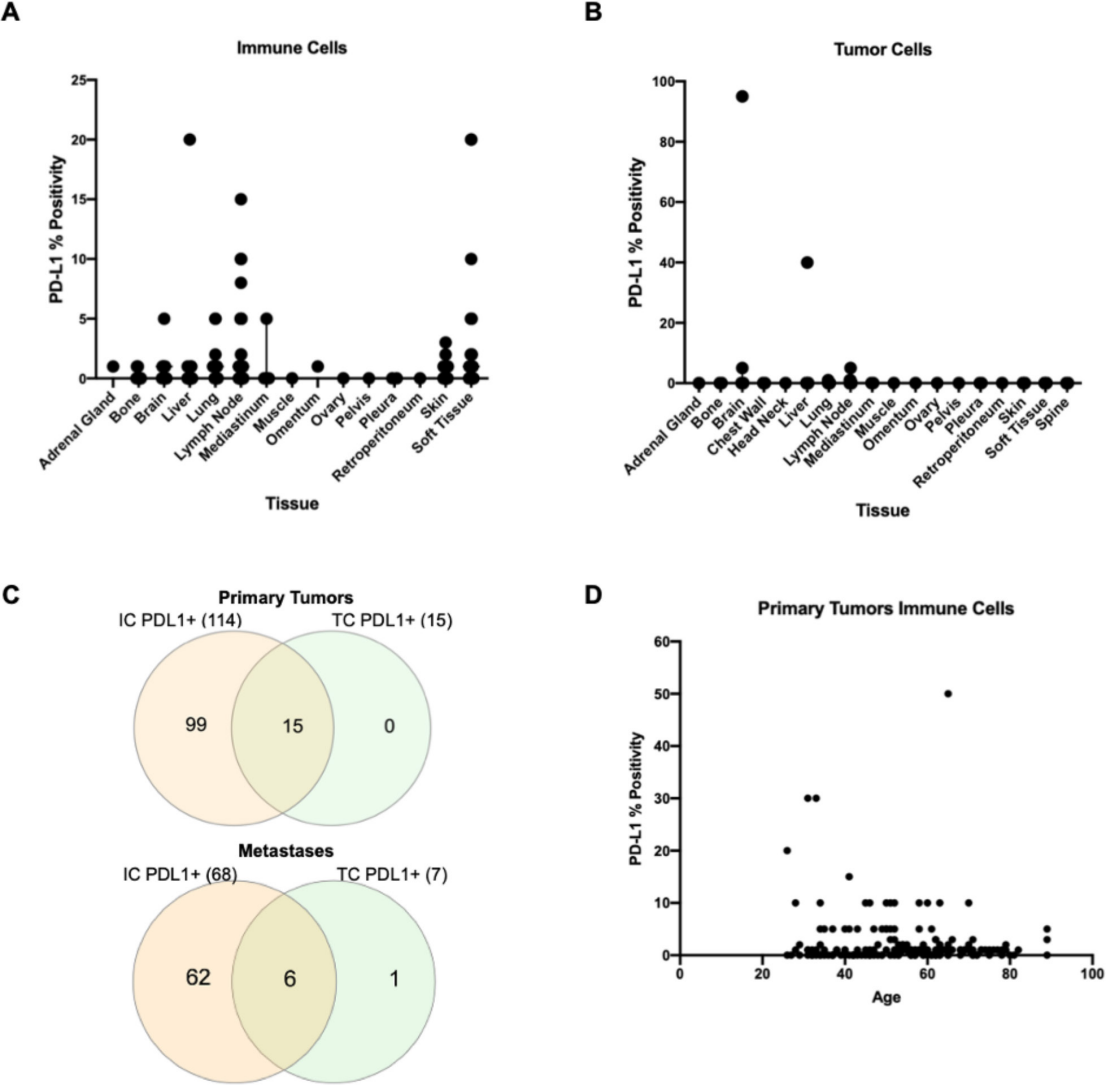
Programmed Death Ligand 1 (PD-L1) expression. (A) PD-L1 percent positivity by IHC on s immune cells by metastatic location. (B) PD-L1 percent positivity by IHC on tumor cells by metastatic location. (C) Venn diagrams of PD-L1 positive ICs (left) and TCs (right) in breast primaries (N=179) and metastatic lesions (N=161). (D) PD-L1 percent positivity on ICs in primary tumors by age (r=0.02, p value=0.7833). IC, immune cells; IHC, immunohistochemistry; TCs, tumor cells.

All patients were female, with a median age of 56 years (range: 26–89 years); 179 samples were from primary tumors and 161 from metastatic lesions, representing 15 different tissue sites. Overall, PD-L1 expression on immune cells was statistically significantly more frequent in primary tumors compared with metastatic sites (63.7% (N=114) vs 42.2% (N=68)), p<0.0001) (tables 1 and 2; figure 1D). When we excluded lymph nodes (locoregional and distant, N=45) from the analysis the overall PD-L1 positivity rate was 39.7%, 95% CI (30.7% to 49.2%) in the rest of the metastatic tissues. We observed substantial heterogeneity in PD-L1 positivity rates across metastatic sites. Lung, soft tissues and lymph node metastases had PD-L1 percent positivity rates that were similar to that of primary tumors, whereas skin, liver and bone metastases had significantly lower PD-L1 percent positivity rates (table 1; figure 1A).

**Table 1:** Sample characteristics and PD-L1 percent positivity on immune cells

Sample type	Total N (%)	N PD-L1 positive (%, 95% CI)*
Primary tumor	179 (52.6)	114 (63.7%, 56.2% to 70.7%)
Metastatic lesion	161 (47.4)	68 (42.2%, 35.1% to 50.9%)
**Sites of metastases**	**N (% of metastatic samples)**	N PD-L1 positive (%, 95% CI)*
Lung	16 (10.0)	11 (68.8%, 41.3% to 90.0%)
Soft tissues	23 (14.3)	15 (65.2%, 42.7% to 83.6%)
Lymph nodes	45 (28.0)	23 (51.1%, 35.8% to 66.3%)
Skin	21 (13.0)	5 (23.8%, 8.22% to 47.2%)
Liver	23 (14.3)	4 (17.4%, 5.00% to 38.8%)
Bone	12 (7.5)	2 (16.7%, 2.10% to 48.4%)
Brain	9 (5.6)	5
Mediastinum	4 (2.5)	1
Pleura	2 (1.2)	0
Muscle	1 (<1)	0
Omentum	1 (<1)	1
Ovary	1 (<1)	0
Pelvis	1 (<1)	0
Retroperitoneum	1 (<1)	0
Adrenal gland	1 (<1)	1

*Percent positivity rates within sample type and 95% CIs are provided only for tissue sites with >10 samples.

PD-L1, Programmed Death Ligand 1.

**Table 2:** Comparison of PD-L1 positivity in primary versus metastatic sites

Tissue	PD-L1+ immune cell	PD-L1− immune cell	P value	PD-L1+ tumor cell	PD-L1− tumor cell	P value
Primary	114	65	0.0001	15	164	0.1313
Metastasis	68	93		7	154	

PD-L1, Programmed Death Ligand 1.

The frequencies of PD-L1 expression on tumor cells was rare in both primary and metastatic cancers and was not significantly different (8.3% (N=15) vs 4.3% (N=7), p=0.13) (table 2). One brain and one liver sample showed high PD-L1 expression on tumor cells, but these cases were also positive for expression in immune cells (figure 1B). There was one lymph node sample that showed PD-L1 positivity (1% positivity) on tumor cells only (figure 1C).

We found no association between PD-L1 expression in immune cells and age.

## Discussion

We found that primary breast cancers showed higher rates of PD-L1 expression than metastases when all metastatic sites were considered together. This result is consistent with findings from earlier studies.^2–4 6^ This overall effect was driven by certain metastatic sites that had substantially lower PD-L1 expression than primary tumors, most notably liver, skin and bone metastases. Other tissue sites, including the lung, soft tissues and lymph nodes, had PD-L1 expression rates similar to primary breast cancers. These observations, overall, are similar to those seen in the IMpassion-130 trial,^2^ which also reported lower average PD-L1 positivity in metastatic biopsies compared with primary tumors (36% vs 44%, p=0.014), and among the metastatic sites, PD-L1 expression was lowest in the liver and highest in the lymph nodes. We noted discordance in our point estimates and those reported by IMpassion-130 trial for positivity rates in skin and soft tissue lesions. These differences are likely due to imprecision in the estimates, in both studies, due to the very low number of cases in these categories (table 3).

**Table 3:** Programmed Death Ligand 1 (PD-L1) positivity in metastatic sites in the Foundation Medicine (FM) database and in the IMpassion-130 data^2^

Sample type	FM total N(%)	FM N PD-L1+ (%)	IMpassion-130 total N(%)	IMpassion-130 N PD-L1+ (%)
Primary tumor	179 (52.6)	114 (63.7)	559 (62)	246 (44)
Metastatic lesion	161 (47.4)	68 (42.2)	342 (38)	123 (36)
**Sites of metastases**	**N (% of metastatic samples**)	**N PD-L1+ (%)**	**IMpassion-130 N (% of metastatic samples)**	**IMpassion-130 N PD-L1+ (%)**
Lung	16 (10.0)	11 (68.8)	54 (15.8)	23 (43)
Soft issues	23 (14.3)	15 (65.2)	36 (10.5)	11 (30)
Lymph nodes	45 (28.0)	23 (51.1)	108 (31.6)	55 (51)
Skin	21 (13.0)	5 (23.8)	18 (5.3)	9 (48)
Liver	23 (14.3)	4 (17.4)	45 (13.2)	6 (13)

Overall, the reason for these apparent organ site-specific differences is unclear. It is possible that technical differences could contribute. Metastatic core needle biopsies may be handled differently than surgical pathology tissues. Differences in durations of warm and cold ischemic time, duration of fixation, pH of the formalin, temperature of paraffin at embedding time and acid decalcification of bone can each influence IHC results.^8 9^ The sensitivity of PD-L1 staining to these preanalytical variables is not well understood. We also recognize that quantifying PD-L1 expression on immune cells in metastatic lymph nodes is challenging due to subjectivity in defining the ‘tumor area’ for immune cell scoring. PD-L1 positivity rates may be inflated in nodal metastases due to the abundance of immune cells. We also recognize the controversy around PD-L1 assay reproducibility and concordance in observed results in the community^10^; however, the centralized, highly quality controlled nature of testing in this study limits the contribution of assay variability to the results. The most likely explanation for the lower and variable PD-L1 expression in metastatic lesions is genuine immunological differences between primary tumors and metastases. Multiple lines of evidence suggest that certain metastatic sites are more immune attenuated than others^11 12^ and primary breast cancers on average have greater immune cell infiltration and higher expression of immune activation markers than metastatic lesions.^3–6^ Preclinical data also suggests that metastatic lesions have a different proportion of monocytes and macrophages that mediate prometastatic functions, including altering antigen presentation, dendritic cell maturation and cytokine signaling.^13^

The clinical implications of the substantial heterogeneity in PD-L1 expression that we observed across metastatic sites are not yet understood. At a practical level, one could conclude that to maximize the opportunity to receive immune checkpoint therapy, PD-L1 staining should be performed on the primary tumor and selected metastatic sites, including lung, soft tissue and possibly lymph node metastases, when available. These results also raise the possibility that response to immune therapy could depend on the location and the PD-L1 positivity of the metastatic site. Limited current experience in breast cancer is not sufficient to correlate organ site-specific tumor response with PD-L1 expression in metastases, but as more patients receive treatment, this could be examined in the future. It should also be noted that patients from IMpassion-130 trial with liver metastasis also benefited from atezolizumab. On a few occasions, we observed higher PD-L1 expression on tumor cells than on immune cells. We also noticed that very rarely, only the tumor cells stain positive for PD-L1 (one case with PD-L1 positive tumors cells in a lymph node). Under the current FDA approval, these tumor-only positive patients are not eligible for immune checkpoint therapy. Even if they represent a small population, it would be important to study immunotherapy efficacy in these patients.
